# Association of *Phlebotomus guggisbergi* with *Leishmania major* and *Leishmania tropica* in a complex transmission setting for cutaneous leishmaniasis in Gilgil, Nakuru county, Kenya

**DOI:** 10.1371/journal.pntd.0007712

**Published:** 2019-10-18

**Authors:** Barrack O. Owino, Damaris Matoke-Muhia, Yasser Alraey, Jackline Milkah Mwangi, Johnstone M. Ingonga, Philip M. Ngumbi, Aitor Casas-Sanchez, Alvaro Acosta-Serrano, Daniel K. Masiga

**Affiliations:** 1 International Centre of Insect Physiology and Ecology, Nairobi, Kenya; 2 Department of Vector Biology, Liverpool School of Tropical Medicine, Liverpool, United Kingdom; 3 Centre for Biotechnology Research and Development, Kenya Medical Research Institute, Nairobi, Kenya; 4 Department of Parasitology, Liverpool School of Tropical Medicine, Liverpool, United Kingdom; 5 King Khalid University, Medical Science College, Abha City, Kingdom of Saudi Arabia; Fundaçao Oswaldo Cruz, BRAZIL

## Abstract

**Background:**

*Phlebotomus (Larroussius) guggisbergi* is among the confirmed vectors for cutaneous leishmaniasis (CL) transmission in Kenya. This scarring and stigmatizing form of leishmaniasis accounts for over one million annual cases worldwide. Most recent CL epidemics in Kenya have been reported in Gilgil, Nakuru County, where the disease has become a public health issue. However, little is known about the factors that drive its transmission. Here, we sought to determine the occurrence, distribution and host blood feeding preference of the vectors, and to identify *Leishmania* species and infection rates in sandflies using molecular techniques. This information could lead to a better understanding of the disease transmission and improvement of control strategies in the area.

**Methodology/ Principal findings:**

An entomological survey of sandflies using CDC light traps was conducted for one week per month in April 2016, and in June and July 2017 from five villages of Gilgil, Nakuru county; Jaica, Sogonoi, Utut, Gitare and Njeru. Sandflies were identified to species level using morphological keys and further verified by PCR analysis of *cytochrome c oxidase subunit I (COI)* gene. Midguts of female sandflies found to harbour *Leishmania* were ruptured and the isolated parasites cultured in Novy-MacNeal-Nicolle (NNN) media overlaid with Schneider’s insect media to identify the species. *Leishmania* parasite screening and identification in 198 randomly selected *Phlebotomus* females and parasite cultures was done by PCR-RFLP analysis of *ITS1* gene, nested kDNA-PCR and real-time PCR-HRM followed by sequencing. Bloodmeal source identification was done by real-time PCR-HRM of the vertebrate *cytochrome-b* gene. A total of 729 sandflies (males: n = 310; females: n = 419) were collected from Utut (36.6%), Jaica (24.3%), Sogonoi (34.4%), Njeru (4.5%), and Gitare (0.1%). These were found to consist of nine species: three *Phlebotomus* spp. and six *Sergentomyia* spp. *Ph*. *guggisbergi* was the most abundant species (75.4%, n = 550) followed by *Ph*. *saevus sensu lato* (11.3%, n = 82). Sandfly species distribution across the villages was found to be significantly different (p<0.001) with Jaica recording the highest diversity. The overall *Leishmania* infection rate in sandflies was estimated at 7.07% (14/198). Infection rates in *Ph*. *guggisbergi* and *Ph*. *saevus s*.*l*. were 9.09% (12/132) and 3.57% (2/56) respectively. *L*. *tropica* was found to be the predominant parasite in Gilgil with an overall infection rate of 6.91% (13/188) in *Ph*. *guggisbergi* (n = 11) and *Ph*. *saevus s*.*l*. (n = 2) sandflies. However, PCR analysis also revealed *L*. *major* infection in one *Ph*. *guggisbergi* specimen. Bloodmeal analysis in the 74 blood-fed sandflies disclosed a diverse range of vertebrate hosts in *Ph*. *guggisbergi* bloodmeals, while *Ph*. *saevus s*.*l*. fed mainly on humans.

**Conclusions/ Significance:**

The high infection rates of *L*. *tropica* and abundance of *Ph*. *guggisbergi* in this study confirms this sandfly as a vector of *L*. *tropica* in Kenya. Furthermore, isolation of live *L*. *tropica* parasites from *Ph*. *saevus s*.*l*. suggest that there are at least three potential vectors of this parasite species in Gilgil; *Ph*. *guggisbergi*, *Ph*. *aculeatus* and *Ph*. *saevus s*.*l*. Molecular identification of *L*. *major* infections in *Ph*. *guggisbergi* suggested this sandfly species as a potential permissive vector of *L*. *major*, which needs to be investigated further. Sandfly host preference analysis revealed the possibility of zoonotic transmissions of *L*. *tropica* in Gilgil since the main vector (*Ph*. *guggisbergi*) does not feed exclusively on humans but also other vertebrate species. Further investigations are needed to determine the potential role of these vertebrate species in *L*. *tropica* and *L*. *major* transmission in the area.

## Author summary

Leishmaniases are diseases caused by *Leishmania* parasites and transmitted through the bites of infected female sandflies during blood feeding. In Kenya, two main forms of the disease exist; visceral (VL) and cutaneous leishmaniasis (CL). Most recent CL epidemics in Kenya have been reported in Gilgil, Nakuru county, where the disease has become a public health problem. However, little is known about the factors that drive its transmission in the area. In this study, we investigated the occurrence, distribution, host preference and *Leishmania* infection rates in sandflies collected from Gilgil using molecular tools. This could lead to a better understanding of the disease and improvement of control strategies in the area. Our results revealed a high infection rate of *L*. *tropica* in two sandfly species (*Ph*. *guggisbergi* and *Ph*. *saevus s*.*l*.), which may indicate a high circulation level of this parasite in the area. However, we also identified *L*. *major* infection from *Ph*. *guggisbergi* species by PCR. Analysis of sandfly bloodmeal sources showed that *Ph*. *guggisbergi* fed on a diverse range of vertebrates including goats, rock hyraxes, rabbits, wild pigs and rodents whereas *Ph*. *saevus s*.*l*. fed mainly on humans. The potential of *Ph*. *guggisbergi* as a vector of *L*. *major* and whether other vertebrate hosts play a role in maintaining *Leishmania* parasites in this CL endemic area needs further investigation.

## Introduction

Human leishmaniases are caused by protozoan parasites of the *Leishmania* genus and transmitted through infective bites of haematophagous female sandflies belonging to the *Phlebotomus* (Old World) and *Lutzomyia* (New World) genera [1]. The disease is among the world’s most neglected tropical diseases (NTDs) occurring in the tropics, sub-tropics and southern parts of Europe [2]. According to the World Health Organization [3] and previous reports [1,4], leishmaniasis is endemic in nearly a hundred low and middle-income countries where there are approximately 350 million people at risk. Furthermore, it is estimated that 2 million cases of the disease occur annually, and the current number of infections is about 12 million [1,5].

There are three main forms of leishmaniasis, influenced mainly by the host immune response and the infecting *Leishmania* spp.: visceral (VL), cutaneous (CL) and mucocutaneous leishmaniasis (MCL) [6,7]. In Kenya, both CL and VL are endemic particularly in the eastern, north-eastern and Rift Valley regions [8,9]. In the Rift Valley alone, Njau (2010) reported more than 50 cases of CL especially in Gilgil during the 2009 epidemics [9]. The disease has since become a public health problem in the area, and it is increasing in geographical coverage. However, little is known about the factors that drive the transmission.

Despite the increasing leishmaniasis research in Kenya, much of these studies have focused on VL owing to its life-threatening nature [4], favouring CL to continue spreading in the background. This is aggravated by the lack of data on CL prevalence country-wide [10–12]. Although CL lesions caused by some parasite species are known to heal without treatment [13], the healing process takes several months or years resulting in deeply stigmatizing and long-term scars which are located mainly on the face [4,13]. This highlights the need for increased efforts to control CL and to bridge the continued psychological morbidity associated with its scars.

The few intermittent CL reports in Kenya indicate that the disease is caused by three distinct species of *Leishmania* parasites: *L*. *major*, *L*. *tropica* and *L*. *aethiopica* [9,10]. *L*. *tropica* was first identified in the central and Rift Valley regions of Kenya [8,10] while *L*. *major* is known to occur in the lowland areas of Baringo and Kitui counties [8,14]. *L*. *aethiopica*, on the other hand, has been reported in the mountainous regions such as Mount Elgon and the Rift Valley escarpments [14]. The sandflies *Ph*. *duboscqi*, *Ph*. *guggisbergi* and *Ph*. *pedifer* have been identified as the vectors of *L*. *major*, *L*. *tropica* and *L*. *aethiopica* respectively [15–17]. *Ph*. *duboscqi* has been shown to exhibit limited distribution, found mainly in small foci of Baringo county in the Rift Valley region [18] whereas *Ph*. *guggisbergi* and *Ph*. *pedifer* are mostly found in caves in various parts of Kenya [16,18]. Small rodents and rock hyraxes (*Procavia capensis*) have been identified as the main reservoir hosts of *L*. *major* [18] and *L*. *tropica* [10,18] respectively. On the other hand, rock hyrax (*Procavia capensis*), tree hyrax (*Dendrohyrax arboreus*) and the giant rat (*Cricetomys gambianus*) have been implicated as the reservoirs of *L*. *aethiopica* in Kenya [18,19].

Sandflies transmit *Leishmania* parasites during feeding; they are ingested with the bloodmeal or regurgitated from an infected sandfly into the host. Since sandflies may feed on a variety of vertebrate hosts which may carry different species of *Leishmania* parasites, identification of the sandfly species and their bloodmeal sources is crucial in incriminating potential vectors, understanding the disease transmission dynamics and identifying the potential ecological reservoirs, which is useful data in developing appropriate disease control and response strategies [20,21].

Demonstration of *Leishmania* parasites in the vectors is a prerequisite for vector incrimination. This is frequently based on the examination of the midguts of dissected female sandflies for the presence of the parasites under a microscope, followed by isoenzyme characterisation of the infecting parasite to the species level [22]. However, the sensitivity of microscopy reduces in the case of low parasitaemia and most sandflies which are positive for *Leishmania* parasites are frequently missed. In recent years, PCR-based molecular methods with a high degree of sensitivity and specificity have been increasingly employed to identify and characterise *Leishmania* parasites to the species level in reservoir hosts and vectors [23]. Indeed, highly sensitive and specific diagnostic procedures are needed for the correct identification and characterisation of the various CL parasites, whose geographical distribution may overlap. This is particularly important when designing disease control and management strategies as some *Leishmania* species have been shown to be resistant to the antileishmanial drugs [8,11,24].

In this study, we combined taxonomic keys with molecular tools to identify sandfly species with potential in transmitting CL parasites in Gilgil, Nakuru county, Kenya. We also applied different molecular assays to identify and characterise the circulating *Leishmania* parasites in the area. To determine the potential animal reservoirs of CL parasites, we identified the bloodmeal sources of engorged sandflies using molecular tools.

## Materials and methods

### Ethical considerations

This study was approved by the Kenya Medical Research Institute (KEMRI), Scientific and Ethics Review Unit (SERU), protocol number (SERU/CBRD/174/3608). Data on CL cases were derived from routine surveillance of the Ministry of Health and local health facilities in Gilgil sub-county, through interactive discussions. Verbal informed consent was sought from homeowners and village elders in order to collect sandflies near homesteads and in private lands a day before trapping.

### Study area

**Fig 1:**
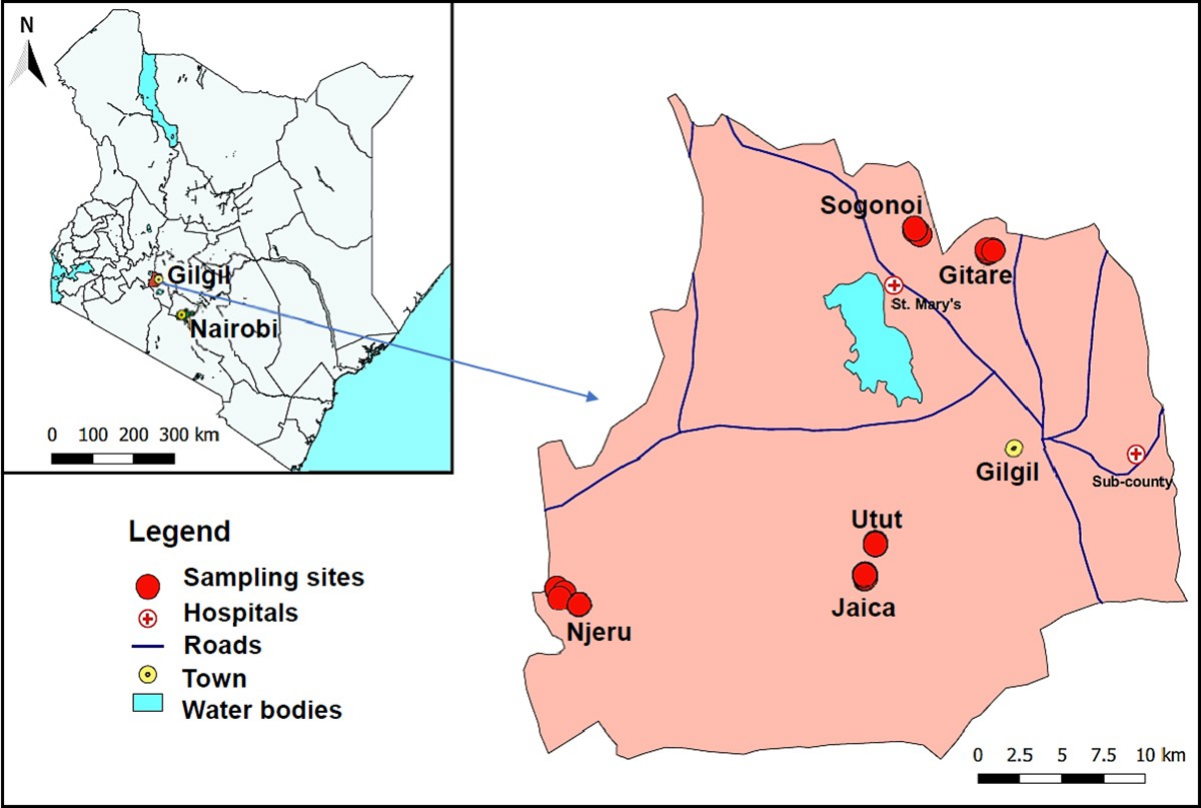
Map of Kenya (inset) and Gilgil Sub-county showing the location of the trapping sites. Gilgil covers an area of approximately 1348.4 Km^2^ and has a population of 138,448 people. Lowest temperatures are experienced in June and July while the highest temperatures occur from December to February. The map was designed using QGIS (v 3.0.3).

An entomological survey of sandflies was conducted for one week per month in April 2016 and in June and July 2017 in five villages of Gilgil sub-county (0°29’32.19” S; 36°19’2.28” E) (Fig 1). This area was selected based on the increase in suspected CL cases among the people seeking medication at health facilities in Gilgil. Since most of the cases were from the larger Utut forest, a historically endemic zone for CL, we selected two villages; Utut and Jaica from this area. Gitare, Njeru and Sogonoi were selected based on reports of new cases. Gilgil receives an average annual rainfall estimated between 700–760 mm while annual mean temperature range is between 24°C and 29.3°C [25]. The altitude ranges of the sampling sites in the five villages were; 1900–1930 m in Jaica and Utut, 2050–2250 m in Njeru, 2150–2250 m in Sogonoi and 2300–2400 m in Gitare.

All the study sites had numerous fault scarp ranges, rock crevices and caves which are potential habitats for sandflies and rock hyraxes (*Procavia capensis*) [10,16,18]. The areas consisted mainly of alternating subsistence farmlands, private ranches and diverse vegetation with considerable wildlife population [10]. Domesticated animals at the sites included cattle (*Bos taurus*), goat (*Capra hircus*), sheep (*Ovis aries*), chicken (*Gallus gallus*) and dogs (*Canis familiaris*) whereas wildlife consisted mainly of rock hyraxes (*Procavia capensis*) which were frequently found around homesteads.

### Sample size estimation and sandfly sampling

In Kenya, studies estimating the prevalence of *Leishmania* infection in the vectors are scarce. To have enough samples for this study, we calculated our sample size assuming an expected prevalence of 50%. Therefore at 95% confidence level and 5% precision, we estimated that a minimum of 385 sandflies was required [26].

Fourteen CDC miniature light traps (John W. Hock Co., Gainesville, FL, USA) were used to sample sandflies in all the study villages. Although light traps and sticky paper traps are the most widely used trapping methods for the host-seeking females and resting populations respectively [27], attempts to use sticky paper traps were unsuccessful due to the occasional rains and cold temperatures and were omitted in the subsequent trapping nights. The traps were placed inside houses and in domestic (livestock shades) and peri-domestic (areas within and around homestead e.g. rodent burrows) sites likely to harbour sandflies. Since sandflies are known to be low fliers, mostly hopping, we set the traps at about 0.5–1.5 metres above the ground at each of the sites, from 1800 hours to 0630 hours the following day to constitute one trapping night. This was repeated for six consecutive trapping nights during the sampling periods.

### Sandfly dissections and morphological species identification

The collected sandflies were sorted out from other insects and washed in 2% detergent followed by antibiotic and antifungal solutions [28]. For all the sandflies, the head and the third last segments of the abdomen were dissected and mounted in gum chloral hydrate for morphological species identification. For females, the abdominal status (blood fed, unfed, gravid) was recorded and the midgut dissected further for parasite cultivation. The dissected midguts were examined at x400 magnification under an Olympus CX31 compound microscope for the presence of *Leishmania* promastigotes and the number of positive sandflies recorded. Parasites were cultured in NNN medium as described by Perkins *et*. *al* [29]. Since we ruptured the abdomen for parasite isolation, we did not determine the developmental stages of the parasites in individual sandflies. Furthermore, sandfly heads were removed for morphological species identification, hence the stomodeal valve was not examined for the infective forms.

Morphological species identification was based on the external genitalia of males and features of the pharynx, antennae and spermatheca for females using different taxonomic keys [30–33]. *Ph*. *guggisbergi* and *Ph*. *aculeatus* males were identified based on the structure and shape of the aedeagus, as well as the number and clustering pattern of the hairy tufts on the inner surface of the coxite [34]. Females of these species were identified using the structures at the base of the spermathecal ducts and pharyngeal armature [34,35]. The width of the style, the structure of the aedeagus and the features of the coxite lobe were used to identify *Ph*. *saevus* males according to the aforementioned keys and Killick-Kendrick *et*. *al* (1997) [36]. For *Ph*. *saevus* females, features of the spermatheca, spermathecal ducts, pharyngeal armatures and length of the third antennae were used [30,36].

The remaining parts of the females after dissections (i.e. the thorax, wings, legs and abdomen) were preserved in 70% ethanol and transported under liquid nitrogen for molecular analyses at the International Centre of Insect Physiology and Ecology (*icipe*) and Liverpool School of Tropical Medicine (LSTM).

### *Leishmania* parasite culture

*Leishmania* parasites were cultured in Novy-MacNeal-Nicolle (NNN) media overlaid with Schneider’s insect media (SIM). In the laboratory, the cultures were kept at 25°C in free SIM and maintained for a total of 14 days to reach the stationary phase with enough parasites for molecular characterisation.

### DNA extraction

We homogenised the remaining parts of the dissected females in 180 μl of buffer ATL (QIAGEN, Hannover, Germany), taking care to avoid contamination between the specimens. Genomic DNA was extracted from the homogenate of each specimen and the parasite cultures using the DNeasy Blood and Tissue Kit (QIAGEN, Hannover, Germany) according to the manufacturer’s recommendations. The extracted DNA was stored at -20°C and used as templates in the subsequent molecular assays.

### Molecular sandfly species identification

Although morphological identification of male species is not very difficult, distinguishing females of some genera based on morphological features is very difficult and requires expertise [37]. To overcome the potential challenges of morphological identification such as cryptic species complexes and phenotypic plasticity, we verified the morphological identifications by PCR analysis of the sandfly mitochondrial *cytochrome c oxidase subunit I* (*COI*) gene as described by Kumar *et*. *al* (2012) using the primers; forward LCO 1490 (5’-GGTCAACAAATCATAAAGTATTGG-3’) and reverse, HCO 2198 (5’-TAAACTTCAGGGTGACCAAAAAATCA-3’) [5]. The 700 bp *COI* amplicons were purified using the QIAquick PCR purification kit (QIAGEN, CA. USA) according to the manufacturer’s protocol and submitted to BioSource (UK) for sequencing under the forward primer. The *COI* chromatograms were edited in Unipro UGENE (v 1.3) to obtain consensus sequences for each sample, followed by sequence similarity search using BLAST (http://www.ncbi.nlm.nih.gov) and multiple sequence alignment using the Clustal W tool in MEGA (v7).

A dendrogram was constructed, taking into account the variations in the sequences, using the maximum-likelihood method [38,39] in agreement with the Kimura 2-parameter model [40]. To estimate the node reliability, we used a bootstrapping value of 1000 replicates and complete deletion method for the gap/missing data. We also included a *COI* sequence of *Lutzomyia umbratilis* (JQ839256.1), a vector of *L*. *guyanensis* in South America, as a reference outgroup to enhance the reliability of the tree.

### *Leishmania* parasite detection and identification

To identify the circulating *Leishmania* parasite species in Gilgil, we screened a random sample of 198 female sandflies (blood-fed = 74; unfed = 124) for the presence of *Leishmania* species. This sample was selected using a stratified random sampling design with probability proportional to size in SPSS (v24). DNA from the individual sandflies were grouped into 19 pools consisting of eight pools of 11 sandflies and eleven pools of 10 sandflies and analysed together with individual DNA from the cultured parasites using *ITS1*-PCR-RFLP and nested *kDNA*-PCR. To enhance the sensitivity of parasite screening and identification, we amplified the samples further by real-time PCR followed by high-resolution melt (HRM) analysis using primers targeting the *Leishmania ITS1* gene. Each of the amplification run was validated for accuracy and sensitivity using reference positive (DNA from *L*. *tropica*: Lv357 strain and *L*. *major*: Friedlin strain) and negative (nuclease-free water) controls.

#### *ITS1*-PCR-RFLP assay

First we screened all the DNA pools and the successfully established parasite cultures by PCR amplification of approximately 320 bp of the *Leishmania internal transcribed spacer 1 (ITS1)* using LITSR (5’-CTGGATCATTTTCCGATG-3’) and L5.8S (5’-TGATACCACTTATCGCACTT-3’) primers according to the protocol described by Schonian *et*. *al* (2003). The 20 μl reaction mixture contained 1x Dream *Taq* buffer with 2 mM MgCl_2_ (Thermo Scientific, USA), 0.25 mM dNTPs mix, 500 nM of each primer, 0.125 U of Dream *Taq* DNA polymerase (Thermo Scientific, USA), 3.5–8.2 ng of DNA template and nuclease free water (Sigma, St. Louis, USA). All the PCR reactions were performed in a SimpliAmp Thermal Cycler (Applied Biosystems, Loughborough, UK). The cycling conditions included an initial denaturation at 98°C for 2 minutes, followed by 35 cycles of denaturation at 95°C for 20 seconds, annealing at 53°C for 30 seconds and extension at 72°C for 30 seconds. This was followed by a final extension at 72°C for 5 minutes. The PCR products were run in 1.5% agarose gel stained with 1x SYBR safe (Thermo Scientific, UK) and visualised in a Gel Doc ^TM^ EZ imager (BIORAD, UK).

Any positive pool and parasite cultures with the expected band size were further analysed individually and subjected to *HaeIII* RFLP analysis without prior purification to identify the infecting *Leishmania* species [41]. The *HaeIII* digestions were carried out in 20 μl reaction volume containing 1x FastDigest buffer (Thermo Scientific, USA), 10 units of *HaeIII* enzyme and 17 μl of PCR products. The reaction conditions included an incubation at 37°C for 2 hours followed by a final incubation at 80°C for 20 minutes to stop the reaction. The *ITS*1-PCR products of the positive samples were further purified using the QIAquick PCR purification kit (Qiagen, CA. USA) and submitted for sequencing under the forward primer to confirm the species.

#### Nested *kDNA* PCR assay

To enhance the sensitivity of *Leishmania* parasite screening and identification [42,43], we further amplified the pooled sandfly DNA using two sets of primers targeting the *Leishmania* kinetoplast DNA. This target was selected due to its presence in high copy number [44]. We used the primers CSB2XF (5’-CGAGTAGCAGAAACTCCCGTTCA-3’) and CSB1XR (5’-ATTTTTCGCGATTTTCGCAGAACG-3’) for the first PCR reaction while 13Z (5’-ACTGGGGGTTGGTGTAAAATAG-3’) and LiR (5’-TCGCAGAACGCCCCT-3’) were used for the second reaction as previously described by Noyes *et*. *al* (1998) [45,46].

#### Real-time PCR-HRM assay

The previous assays did not reveal *Leishmania* infections in most sandfly samples and therefore real-time PCR followed by HRM was applied. To improve the resolution of identifying the *Leishmania* species, the primers (F: 5’-CACGTTATGTGAGCCGTTATCC-3’; R: 5’-GCCTTTCCCACATACACAGC-3’) were manually designed based on the nucleotide alignment of the *ITS1* sequences from *L*. *major* and *L*. *tropica* using Clustal Omega, with a melting temperature of 60°C and predicted amplicons of 195 bp for *L*. *major* and 179 bp for *L*. *tropica*.

All the reactions were carried out in an HRM capable Agilent Technologies Stratagene Mx3005P real-time PCR thermocycler (Agilent Technologies, Santa Clara, USA). The 20 μl final volume contained 1x Luna Universal qPCR SYBR Green-based master mix (NEB, UK), 500 nM of each primer, 1 μl of the template and nuclease free water (Sigma, St. Louis, USA). Cycling conditions included an initial denaturation at 95°C for 1 minute followed by 40 cycles of denaturation at 95°C for 15 seconds and annealing and extension at 60°C for 30 seconds. HRM was performed by denaturation of the real-time PCR products at 95°C for 1 minute followed by cooling at 50°C for 30 seconds for reannealing and gradually raising the temperature by approximately 0.1°C increments per 2 seconds and recording changes in fluorescence. To estimate the cut-off level for positivity, 198.5 ng of DNA extracted from cultured *L*. *tropica* (Lv357) promastigotes was diluted in water to 1.3 x 10^−9^ ng/μl. From the standard curve generated using 1 μl of DNA from each diluent, samples with cycle threshold (Ct) level of < 33 were treated as positive. To confirm the parasite identities, the positive samples were further processed as described in the previous sections and submitted for sequencing under the forward primer.

### Host blood meal identification

To identify the bloodmeal sources of the blood-fed sandflies, we amplified a region of the vertebrate mitochondrial *cytochrome b* (*CYT-B*) gene using universal *CYT-B* -specific primers as described previously by Omondi *et al*. (2015) [21]. Without stopping the reaction, the established 383 bp PCR products were resolved by HRM. All the real-time PCR-HRM reactions were performed in Rotor-Gene Q real-time PCR thermocycler (QIAGEN, Hannover, Germany) using *CYT-B* forward (5’-CCCCTCAGAATGATATTTGTCCTCA-3’) and *CYT-B* reverse (5’-CATCCAACATCTCAGCATGATGAAA-3’) primers. DNA extracted from known vertebrate blood samples were used as positive controls. These included animals commonly found in the study area such as goat, sheep, cow and hyrax. We also included a human blood sample obtained from a volunteer in *icipe* and Swiss mouse, rabbit and rat blood sourced from *icipe’s* animal rearing unit. Blood from the other animals was sourced from a butchery.

Identification of the bloodmeal sources was done by comparing the melting profiles of the samples to those of positive controls. Samples whose profiles did not match those of the controls were purified using ExoSAP-IT (USB Corporation, Cleveland, OH) DNA purification kit, according to the manufacturers’ instructions and submitted for sequencing under the forward primer. The *CYT-B* chromatograms were edited in Unipro UGENE (v 1.3) and queried against the GenBank database using the NCBI’s BLASTn. The top hit vertebrate species with the lowest e-value and homology cut-off values of 70%-100% were selected as the most likely sandfly bloodmeal hosts.

### Statistical analysis

Details of all the collected sandflies including the trapping site, GPS location, collection date and trap number were entered in Microsoft Excel (2016) by two different field assistants and compared for consistency using SPSS (v24). Since only one sandfly was collected from Gitare, we excluded this village in our diversity analysis. Descriptive statistics were used to determine the distribution pattern and frequency of each sandfly species per village. Species abundance was determined as the quantitative counts per village. Differences in the species distribution were analysed using the Kruskal-Wallis test. We calculated the sex ratio of all the species as; (number of males/number of females) x 100. Differences in sex ratios were determined using the chi-square test. We calculated Shannon Weiner index (H’), followed by computing its reverse (exp^H’^) to obtain the effective number of species per village. The effective number of species is the number of equally-common species required to give a particular value of an index [47–49]

## Results

### Sandfly species composition and diversity

A total of 729 sandflies (419 females and 310 males) were collected and identified to species level using morphological keys. Of the females, 74 (17.7%) were fully or partly engorged while the rest were unfed. Three *Phlebotomus* species belonging to two subgenera and six *Sergentomyia* spp. were identified (Table 1). Species of the *Paraphlebotomus* sub-genus included *Ph*. *saevus s*.*l*. while those of the *Larroussius* subgenus were *Ph*. *guggisbergi* and *Ph*. *aculeatus*. The *Sergentomyia* species included; *S*. *thomsoni*, *S*. *bedfordi*, *S*. *schwezi*, *S*. *antennatus*, *S*. *squamipleuris* and *S*. *adami*. *Ph*. *guggisbergi* was the most abundant and ubiquitous species (75.4%) (S1 Fig) followed by *Ph*. *Saevus s*.*l*. (11.3%) whereas *Ph*. *aculeatus* represented 5.6% of the collected samples. Sandfly density was highest in Utut village followed by Sogonoi while Gitare had the lowest density.

**Table 1:** Sandfly density, relative abundance (%) and sex ratios in Gilgil.

Species	Jaica F M D			Njeru F M D			Sogonoi F M D			Utut F M D			Gitare F M D			Total F M		Abundance n (%)
*Ph*. *guggisbergi*	66	40	1.893	5	0	0.357	132	119	5.976	111	76	4.452	1	0	0.036	315	235	550 (75.4)
*Ph*. *saevus s*.*l*.	20	8	0.500	11	9	1.429	-	-	-	26	8	0.810	-	-	-	57	25	82 (11.3)
*Ph*. *aculeatus*	2	5	0.125	-	-	-	-	-	-	7	27	0.810	-	-	-	9	32	41 (5.6)
*S*. *bedfordi*	5	0	0.089	6	2	0.571	-	-	-	5	4	0.214	-	-	-	16	6	22 (3.0)
*S*. *schwezi*	13	5	0.321	-	-	-	-	-	-	1	0	0.024	-	-	-	14	5	19 (2.6)
*S*. *thomsoni*	6	3	0.161	-	-	-	-	-	-	0	1	0.024	-	-	-	6	4	10 (1.4)
*S*. *adami*	0	3	0.054	-	-	-	-	-	-	-	-	-	-	-	-	0	3	3 (0.4)
*S*. *squamipleuris*	1	0	0.018	-	-	-	-	-	-	-	-	-	-	-	-	1	0	1 (0.1)
*S*. *antennatus*	-	-	-	-	-	-	-	-	-	1	0	0.024	-	-		1	0	1 (0.1)
**Total**	**113**	**64**	**3.161**	**22**	**11**	**2.357**	**132**	**119**	**5.976**	**151**	**116**	**6.357**	**1**	**0**	**0.036**	**419**	**310**	**729 (100)**

F: females; M: males; D: density (number of sandflies/trap/night); *s*.*l*.: *sensu lato*.

**Fig 2:**
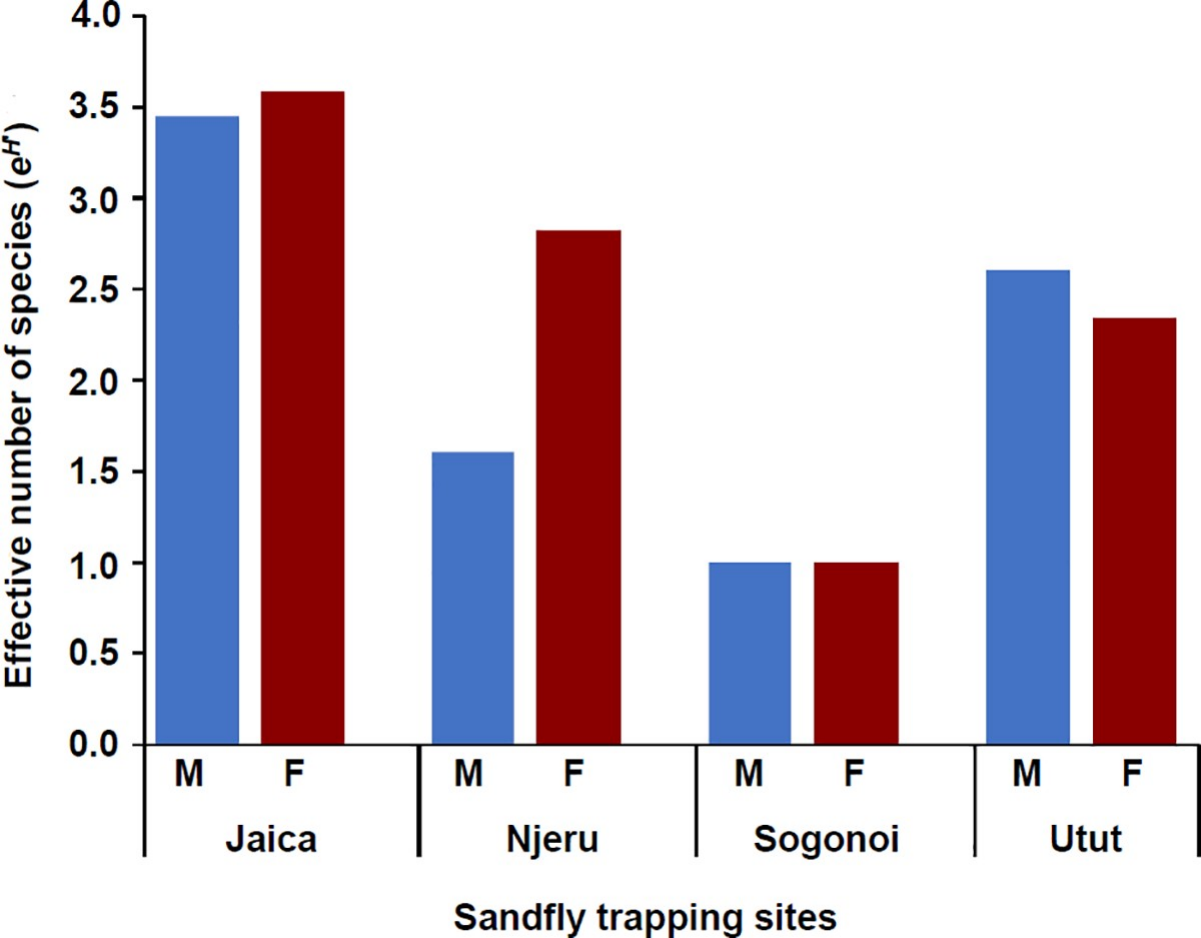
Sandfly species diversity across the sampling sites in Gilgil. M: male; F: female.

The overall distribution of sandfly species across the villages was found to be significantly different (p<0.001). The numbers of females collected were significantly higher than that of males (χ^2^ (1, n = 729) = 16.30, p<0.001) with an overall sex ratio (males/females) of 1:1.35. The highest diversity of sandflies was recorded in Jaica followed by Utut while Sogonoi had the lowest (Fig 2). Female species in Jaica were almost four times as diverse as those of Sogonoi.

### Molecular identification of sandfly species

**Fig 3:**
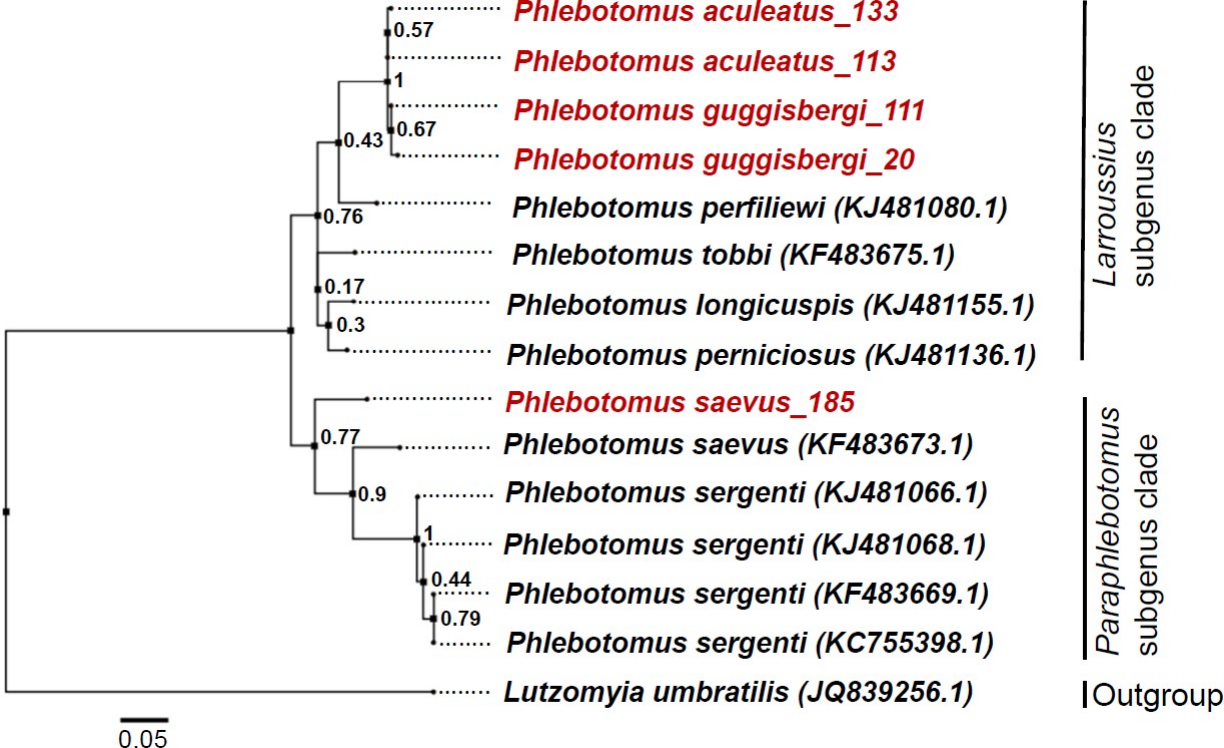
Phylogenetic analysis of the sandfly mitochondrial *COI* sequences by Maximum Likelihood method. The analysis was performed in MEGA v.7 based on the Kimura 2-parameter model. Bootstrap support values based on 1000 replicates are shown at the nodes. Other reference phlebotomine species belonging to different subgenera were included for comparison. The tree is drawn to scale, with branch lengths measured in the number of substitutions per site. Sample identities are shown in red while sequences retrieved from the GenBank are indicated in black. Representative *CO1* sequences for each species are available in the GenBank under accession numbers: MK169221-MK169223.

Randomly selected *COI* sequences (n = 5) of three different morphologically identified female *Phlebotomus* species were verified based on their molecular characteristics. Alignments of *Ph*. *saevus s*.*l*. sequence (GenBank accession number MK169223) revealed 87% similarity to the Israel specimen of the same species retrieved from the GenBank (accession: KF483673.1). These were further grouped into the *Paraphlebotomus* clade by phylogenetic analysis thus confirming the morphological identifications (Fig 3). BLAST search results of *Ph*. *guggisbergi* (MK169221) and *Ph*. *Aculeatus* (MK169222) sequences showed a high degree of similarities (85–92%) to those of *Ph*. *perfiliewi* (KJ481080.1), *Ph*. *longicuspis* (KJ481155.1), *Ph*. *perniciosus* (KJ481136.1) and *Ph*. *tobbi* (KF483675.1). However, they were separated into different sub-branches by phylogenetic analysis. *Ph*. *guggisbergi* and *Ph*. *aculeatus* were found to be more closely related to each other than the other species.

All the species under the *Larroussius* subgenus clustered together with two sub-branches; one including *Ph*. *longicuspis* and *Ph*. *perniciosus* and the other including *Ph*. *aculeatus* and *Ph*. *guggisbergi*. For the *Paraphlebotomus* subgenus, *Ph*. *sergenti*, and *Ph*. *saevus* clustered together but with different sub-branches. *Ph*. *saevus s*.*l*. sequence from Kenya was separated from *Ph*. *saevus* specimen from Israel. This could suggest the presence of many lineages in *Ph*. *saevus* populations separated by geographic distance across the species distribution range. Although *Ph*. *perfiliewi*, *Ph*. *perniciosus*, *Ph*. *tobbi*, and *Ph*. *longicuspis* belong to the same subgenus (*Larroussius*) as *Ph*. *guggisbergi* and *Ph*. *aculeatus*, they are mainly found in the Mediterranean region where they are associated with the transmission of *L*. *infantum* [37,43]. In contrast, *Ph*. *guggisbergi* and *Ph*. *aculeatus* are restricted to East Africa, particularly in Kenya (both species) and Ethiopia (*Ph*. *aculeatus*) [30]. The difference in geographical and climatic conditions could explain the variability in the sequences of these species.

### *Leishmania* parasite detection and identification

#### *Leishmania* infection rates in sandflies

Seven of the 419 morphologically identified female sandflies were found to harbour *Leishmania* promastigotes based on microscopy. These were of two species; *Ph*. *guggisbergi* (n = 5) and *Ph*. *saevus s*.*l*. (n = 2). Of the seven isolated parasites, only two were successfully established in cultures for molecular analysis. Most of the infected sandflies were from Sogonoi (n = 4) while the rest were from Utut (n = 2) and Jaica (n = 1). Seven additional *Ph*. *guggisbergi* samples were found to be positive for both *L*. *tropica* (n = 6) and *L*. *major* (n = 1) based on real-time PCR-HRM and sequencing of the PCR-HRM amplicons. Infection rates in *Ph*. *guggisbergi* and *Ph*. *saevus s*.*l*. were 9.09% (12/132) and 3.57% (2/56) respectively. The overall *Leishmania* infection rate in female sandflies in the study area was 7.07% (n = 14/198).

#### *ITS1*-PCR-RFLP

**Fig 4:**
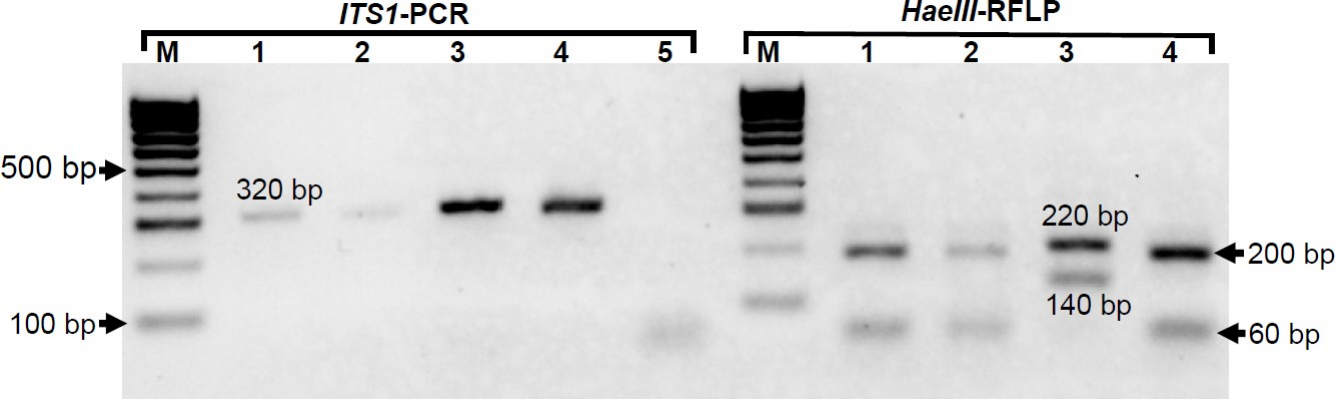
*Leishmania* parasite identification by *ITS1*-PCR-RFLP. M: 100 bp ladder;1 and 2: *Leishmania* spp. isolated from sandflies; 3 and 4: *L*. *major* (Friedlin strain) and *L*. *tropica* (Lv357 strain) positive controls; 5: negative control. Molecular sizes of RFLP product for each species are shown; *L*. *major* (220 and 140 bp); *L*. *tropica* (200 and 60 bp).

None of the pooled sandfly DNA was found to be positive for *Leishmania* parasites using *ITS1*-PCR-RFLP. This could be due to the reduced sensitivity of the *ITS1* target, especially in the DNA pools where the potentially positive samples have been diluted. However, *ITS1*-PCR products of the two successfully established parasites isolated from sandflies produced bands of approximately 320 bp. Digestion of the products with *HaeIII* gave an RFLP pattern characteristic of *L*. *tropica* for all the two isolates (Fig 4).

#### Nested *kDNA*-PCR

Both cultured *Leishmania* isolates produced a 750 bp amplicon, the *L*. *tropica* specific band, confirming the *ITS1*-PCR-RFLP results (S2 Fig). However, none of the pooled DNA was found to be positive for *Leishmania* spp. and were further subjected to *ITS1*- real-time PCR followed by HRM.

#### Real-time PCR-HRM

**Fig 5:**
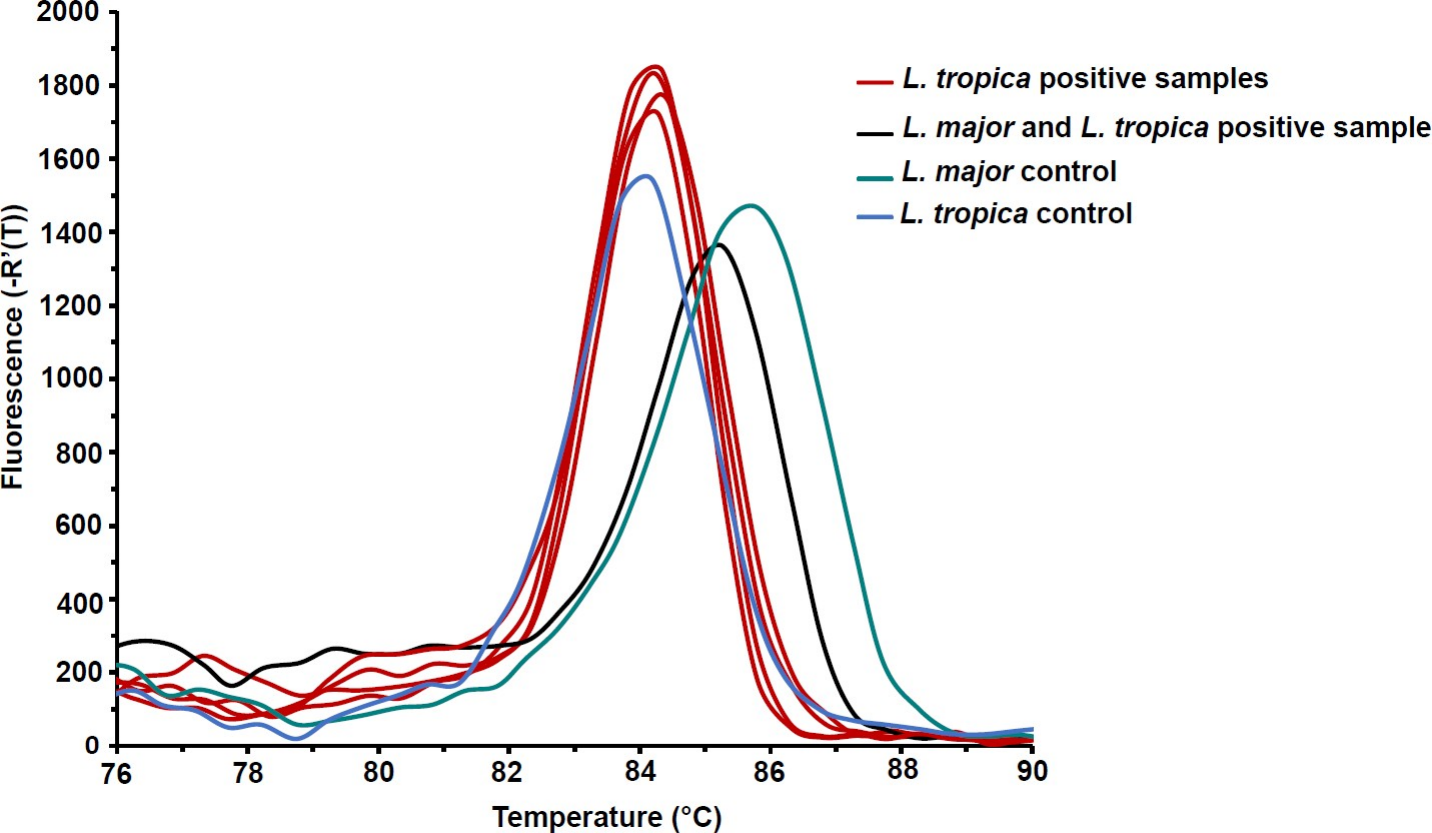
Real-time PCR-HRM analysis of *Leishmania* infection in female sandflies. A derivative dissociation curve of 179 bp *L*. *tropica* (Tm = 84.2°C) and 195 bp *L*. *major* (Tm = 85.8°C) *ITS1*-PCR amplicons. Infection was inferred by comparing the melting profiles of the samples to the controls.

*Leishmania* DNA was detected in three sandfly DNA pools using this method. Confirmation of *Leishmania* spp. in individual sandflies belonging to these pools revealed *L*. *tropica* in six samples belonging to *Ph*. *guggisbergi* species and *L*. *major* in one sample of the same vector (Fig 5). The HRM profile of *L*. *major* found in the naturally infected sandfly varied slightly to that of the control. This variation could be due to disproportionate co-infections with *L*. *major* and *L*. *tropica* where the dominant species is *L*. *major*. Indeed, our preliminary HRM analyses with *L*. *major* and *L*. *tropica* lab strain DNA mixed in 1:1 proportion revealed HRM profiles with an amplicon melting temperature value (Tm = 85.3°C) between that of *L*. *tropica* (Tm = 84.2°C) and *L*. *major* (Tm = 85.8°C) reference controls (S3 Fig). However, the intensity of fluorescence in the disproportionate co-infections varied slightly, with the left tail shifting either towards *L*. *tropica* or *L*. *major* depending on the dominant species. Sequence analysis of this sample revealed superimposed peaks which further suggested co-infection in the sample (S4 Fig).

### Sandfly bloodmeal analysis

**Fig 6:**
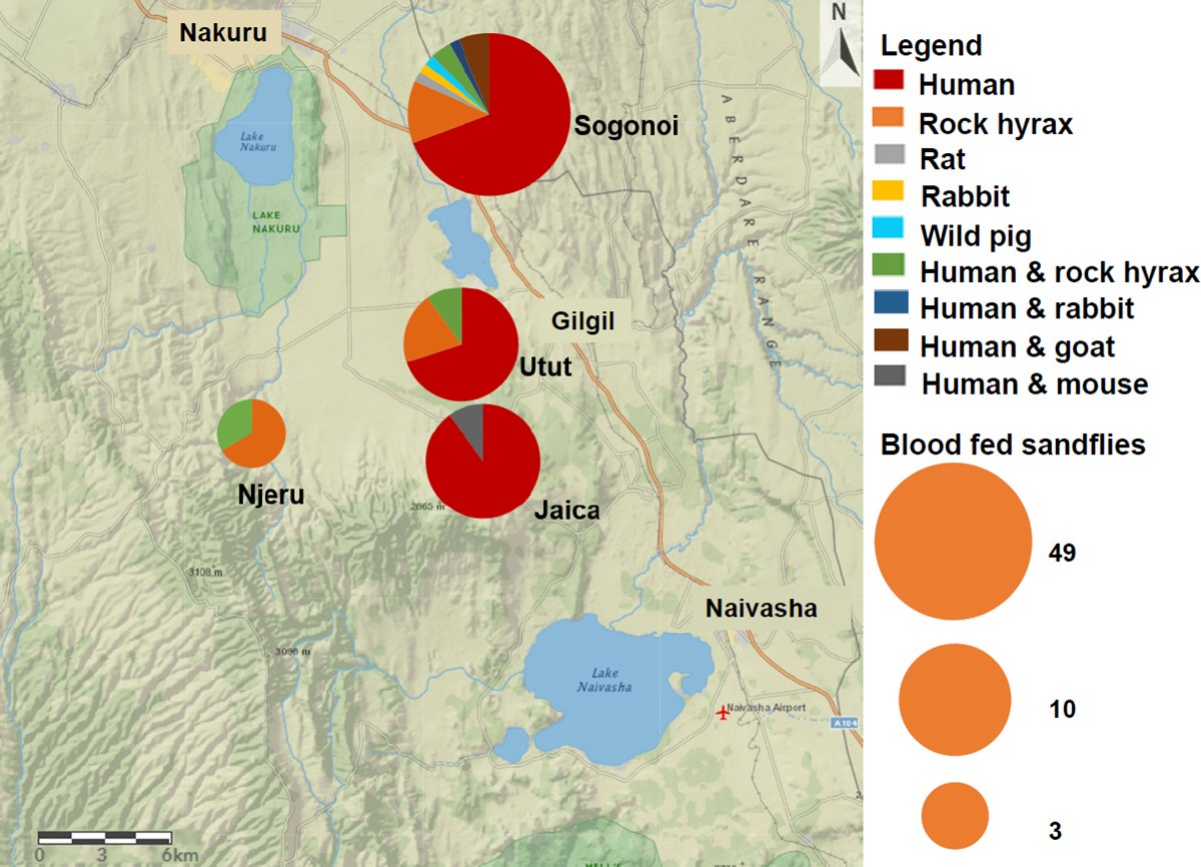
Map of Gilgil showing the proportions of bloodmeal sources per trapping site. The map was designed using ArcGIS Online version.

Presence of potential animal reservoirs was evaluated by analysing the bloodmeal sources of all the 74 fully or partially blood-fed sandflies. The *CYT-B* PCR-HRM and sequencing revealed a variety of vertebrate hosts in sandfly bloodmeals, including humans, domestic, peri-domestic (commonly found around the homestead) and wild animals (Table 2). Humans (*Homo sapiens*) were the predominant sandfly bloodmeal sources constituting 67.57% of pure sandfly bloodmeals, followed by rock hyraxes (*Procavia capensis*) which formed exclusively 13.51%. Bloodmeal sources from domestic animals were mainly from goats (*Capra hircus*) while peri-domestic bloodmeal sources included rats (*Rattus norvegicus*) and mouse (*Mus musculus*). Wild animal hosts included rock hyraxes (*Procavia capensis*), wild rabbits (*Oryctolagus cuniculus*) and wild pigs (*Sus scrofa*). Although humans were the predominant bloodmeal sources in Jaica, Utut and Sogonoi, other vertebrate species were also identified in bloodmeals of sandflies from these areas (Fig 6). In contrast, no human bloodmeal was identified from the analysed sandfly samples collected from Njeru.

**Table 2:** Bloodmeal sources of female sandflies identified by real-time PCR-HRM analysis of *CYT-B*.

						Mixed bloodmeal sources				
species	Human	Hyrax	Rat	Wild Pig	Rabbit	Human & Goat	Human& Rabbit	Human &Hyrax	Human& mouse	UD
*Ph*. *guggisbergi*	46 (8)	9 (3)	1	1	1	3	1	4 (1)	1	2
*Ph*. *saevus s*.*l*.	4 (1)	0	0	0	0	0	0	0	0	0
*S*. *bedfordi*	0	1	0	0	0	0	0	0	0	0
**Total**	**50 (9) (67.57%)**	**10 (3) (13.51%)**	**1 (1.35%)**	**1 (1.35%)**	**1 (1.35%)**	**3 (4.05%)**	**1 (1.35%)**	**4 (1) (5.41%)**	**1 (1.35%)**	**2 (2.70%)**

**UD**: undetermined; the number between the brackets is the amount of blood fed sandflies positive for *Leishmania* spp.

**Fig 7:**
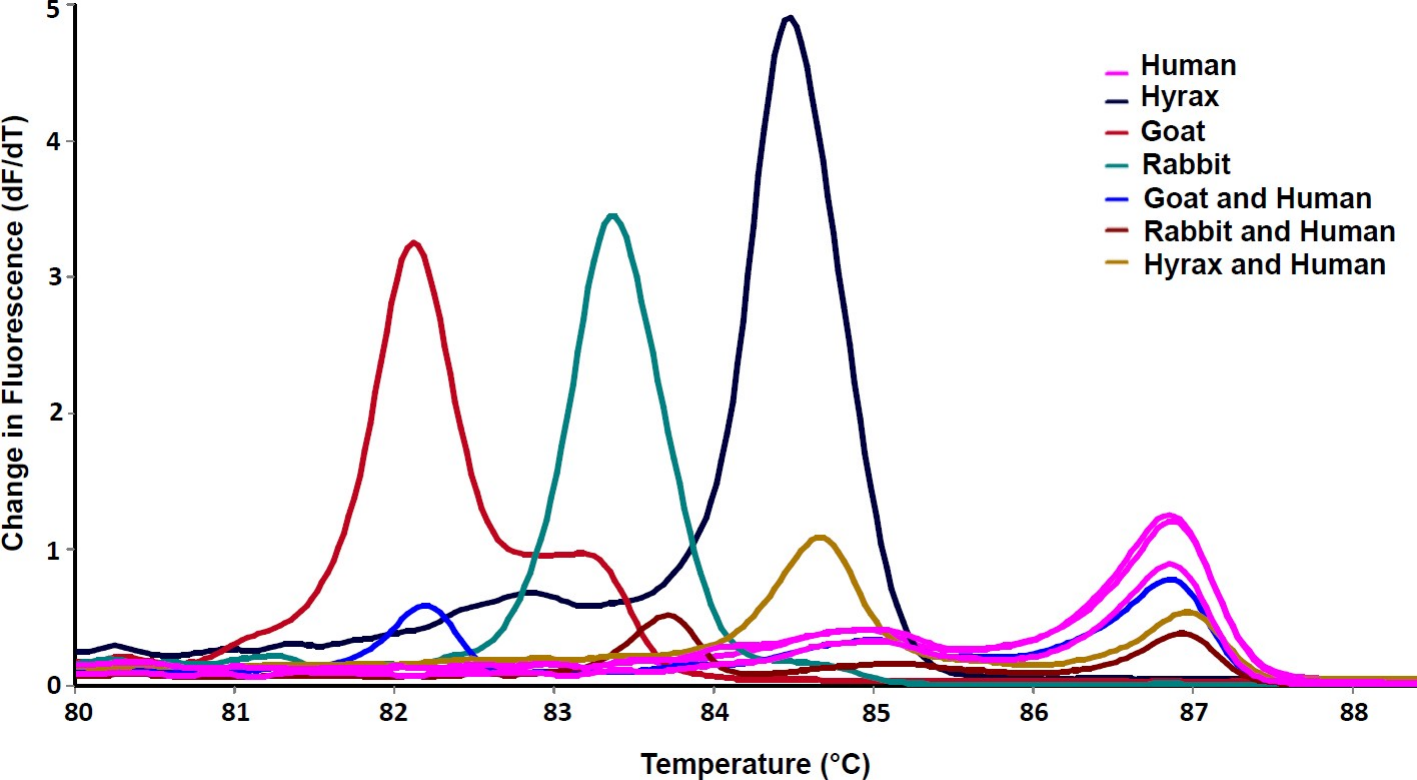
HRM profiles of vertebrate hosts present in sandfly bloodmeals using *CYT-B*. Positive controls are shown in the legend; human, rock hyrax, goat and rabbit. The melting profiles of mixed bloodmeals varied slightly compared to those of pure bloodmeals. Bloodmeal source detection failed in two partially blood-fed sandflies (2.70%). This could be due to the degradation of DNA in the bloodmeal. The *e*-values of BLAST search matches to GenBank sequences, percentage identities and GenBank accessions for some of the exclusive blood-fed sandflies are provided in Table 3.

Mixed bloodmeals were identified based on the presence of HRM profiles with multiple peaks compared to the reference controls (Fig 7). Nine *Ph*. *guggisbergi* species were found to have fed on multiple hosts; rock hyraxes and humans (n = 4), humans and goat (n = 3), human and rabbit (n = 1).

**Table 3:** Vertebrate species represented in sand fly bloodmeals.

Vertebrate species	Sandfly species	GenBank accession	% identity (e-value)
Humans (*Homo sapiens*)	*Ph*. *guggisbergi* *Ph*. *saevus s*.*l*.	KX697544.1 KX697544.1	99 (1*e*-150) 99 (7*e*-150)
Rock hyrax (*Procavia capensis*)	*Ph*. *guggisbergi* *S*. *bedfordi*	D86909.1 D86909.1	92 (3*e*-118) 84 (6*e*-41)
Rabbit (*Oryctolagus cuniculus)*	*Ph*. *guggisbergi*	HQ596486.1	88 (2*e*-90)
Wild pig (*Sus scrofa*)	*Ph*. *guggisbergi*	FM205713.1	98 (5*e*-141)
Rat (*Rattus norvegicus*)	*Ph*. *guggisbergi*	KP233827.1	97 (8*e*-149)

## Discussion

Cutaneous leishmaniasis caused by *L*. *major* and *L*. *tropica* is endemic in many parts of Kenya, particularly in the Rift Valley, Eastern and Central regions [11]. The disease is most prevalent in the central part of Rift Valley especially in Gilgil where the most recent cases have been reported [9]. The objectives of this study were to identify sandfly species with the potential to transmit *Leishmania* parasites and the circulating *Leishmania* spp. responsible for CL occurrence in Gilgil area of Nakuru county. We were also interested in the bloodmeal sources of engorged female sandflies collected from this area. Microscopy has been used for decades as the gold standard test for demonstrating natural *Leishmania* infections in sandflies [50]. However, the sensitivity of this technique reduces with a reduction in parasite loads and most infections are often missed in the vectors. This study provides contemporary data on sandfly species diversity and prevalence of *Leishmania* infections in vectors from Gilgil. Furthermore, it reports for the first time the isolation of *L*. *tropica* from *Ph*. *saevus s*.*l*. and identification of *L*. *major* infections in *Ph*. *guggisbergi*.

Nine sandfly species belonging to the *Phlebotomus* and *Sergentomyia* genera were identified from all the study villages. The *Phlebotomus* species identified represented two out of the five *Phlebotomus* subgenera described in Kenya to date [18]. Sandfly species of the *Larroussius* subgenus included *Ph*. *guggisbergi* and *Ph*. *aculeatus* while that of the *Paraphlebotomus* subgenus was *Ph*. *saevus s*.*l*. In this study, we demonstrated that *Ph*. *guggisbergi* is the predominant sandfly species (75.4%) present in all the five study sites, followed by *Ph*. *saevus s*.*l*. (11.3%). Indeed, we found a significant difference in the overall sandfly species distribution in the area, which could be attributed to the differences in altitude among the sampling sites [16]. This could further explain why only one sandfly was trapped from Gitare, which is situated at the highest altitude. Furthermore, due to logistical constraints, we carried out sampling during the rainy season (April) and the coldest months (June and July) when sandfly activity is expected to be low [16]. Further studies are needed to determine seasonal variations in sandfly densities per village which could unravel areas where high transmission is likely to occur and seasons for sandfly vector control.

The number of female sandflies was found to be higher than that of males with a male/female ratio of 1:1.35. This conforms with other studies in which CDC light traps were used for trapping sandflies. For instance, Mukhwana *et al*. (2018) demonstrated that the number of female sandflies collected using CDC light traps were more than twice the number of males in all their study sites [16]. A possible explanation could be the trapping method which has been shown to be suitable for host-seeking females [27]. Female sandflies require a blood meal for egg development and maturation [20]. Because the mouthparts of male flies are less developed for bloodsucking [51], they tend to have reduced dispersion capacity than females [16]. This could further explain why we did not trap *Ph*. *guggisbergi* males from Njeru, yet it is the most abundant species in Gilgil.

Based on the Shannon-Weiner-index that quantifies the species diversity, the highest diversity of sandflies was recorded in Jaica followed by Utut. These two villages are located in the larger Utut forest, a historically known CL endemic focus [10]. According to the stability hypothesis that diversity gradually reduces from population origin to newly colonised areas [16], though this could also be associated with landscape changes in neighbouring areas as human habitation and other activities. Indeed, this forest supports a wide range of wildlife including the rock hyraxes which have been implicated as the reservoirs for *L*. *tropica* [10,16,18]. The diverse vegetation present in the Utut area possibly attracts people from neighbouring communities into the area for charcoal burning and poles for constructing huts. The difference in species diversity between Utut and the other villages indicates that ecological disturbance due to the encroachment of human activities on sandfly habitats may have resulted in the changes in sandfly distribution and diversity.

Sandfly dispersion is critical to the spread of *Leishmania* parasites. The high abundance of sandflies belonging to the *Phlebotomus* genus in all the study sites could indicate a high risk of CL transmission in the area. Undoubtedly, *Leishmania* infection prevalence in sandflies is one of the indicators of disease transmission intensity [22]. Here, we estimated the overall *Leishmania* infection rates in sandflies to be as high as 7.07% (n = 14/198). A high *Leishmania* infection rate was observed in blood-fed sandflies (6.57%; n = 13) compared to the unfed (0.51%; n = 1). This observation was expected as the unfed sandflies comprise a larger proportion of newly emerged adults that have not acquired the parasites through bloodmeals. This finding further supports those of Ajaoud *et al*., (2015) in which they found high infection rates in the fed sandflies compared to the unfed ones [52].

The high infection prevalence in sandflies and the high vector abundance and diversity could possibly explain the increasing incidences of CL in Gilgil. Isolation of *Leishmania* spp. in five *Ph*. *guggisbergi* and further identification of *L*. *tropica* in six *Ph*. *guggisbergi* species confirms this sandfly as the vector of *L*. *tropica* in Kenya as shown in the previous reports [9,11,17,18]. However, *L*. *major* was also identified from this species, suggesting that it could be a potential permissive vector for both the CL parasites. Indeed, some sandfly species in the *Larroussious* subgenus, for example, *Ph*. *perniciosus* are known permissive vectors in other parts of the world [53]. Although *Ph*. *guggisbergi* has been the most proposed vector of *L*. *tropica* in Kenya, this parasite was also identified from naturally infected *Ph*. *saevus s*.*l*. species. *Ph*. *saevus s*.*l*. belongs to the *Paraphlebotomus* subgenus as *Ph*. *sergenti*, a known vector of *L*. *tropica* in parts of Asia [54] and Africa [52], and was the most abundant species collected from Njeru village where new cases of CL have been reported. Natural infections of *Ph*. *saevus s*.*l*. and *Ph*. *guggisbergi* with *L*. *tropica* and their presence in high abundance suggests that they are the most likely vectors of *L*. *tropica* in Gilgil. Since the head of all females was removed for morphological identifications, we did not assess the percentage of metacyclics at the stomodeal valve of these vectors. Further studies are needed to determine vector competence through the determination of developmental stages in individual field collected sandflies, transmission experiments or *Leishmania* developmental stage-specific gene expression.

*Ph*. *aculeatus* was found to be more closely related to *Ph*. *guggisbergi* by phylogenetic analysis. Although males of these species are not difficult to identify based on morphological features, distinguishing their females may be difficult. Females of the *Larroussius* subgenus exhibit a characteristically long extension of the spermathecal neck [18,37] and most species are indistinguishable based on morphological characters alone. For instance, Absavaran and colleagues demonstrated that females of *Ph*. *major* and *Ph*. *neglectus* were very similar and morphologically indistinguishable [37]. Although sandfly species identification is based majorly on morphological characteristics, supplementing these with molecular tools could help in resolving problems with distinguishing morphologically similar species. The close relationship between *Ph*. *guggisbergi* and *Ph*. *aculeatus* may further implicate the latter species as a probable vector of *L*. *tropica* in Kenya [15,18]. Moreover, identification of live *L*. *tropica* parasites in *Ph*. *saevus s*.*l*. in this CL focus implies that there are at least three *Phlebotomus* spp. transmitting the parasite in Gilgil area: *Ph*. *guggisbergi* (known vector), *Ph*. *aculeatus* (probable vector), and *Ph*. *saevus s*.*l*.

Real-time PCR-based amplification of the *Leishmania ITS1*, followed by HRM, was found to be highly sensitive in identifying *Leishmania* infections in sandflies over the nested *kDNA*-PCR and the *ITS1*-PCR. Furthermore, this technique was highly specific in discriminating between *L*. *major*, *L*. *tropica* and mixed infections based on their melting temperatures. The high sensitivity and specificity of real-time PCR highlight its suitability in screening and diagnosis of CL parasites, especially in endemic regions where multiple *Leishmania* species may coexist [55]. Combining different molecular methods for the epidemiological studies of *Leishmania* in field-caught sandflies is useful for accurate detection and characterisation of the infecting parasites. Direct analysis of infection in field-collected samples may reveal unexpected results including co-infections [52].

Several vertebrate species including humans were found to be fed on by *Ph*. *guggisbergi* based on bloodmeals analysed, whereas *Ph*. *saevus s*.*l*. fed mainly on humans. Although CL due to *L*. *tropica* is frequently regarded as anthroponotic [56], zoonotic transmission of this parasite has been reported in other countries [52]. Since the main vector, *Ph*. *guggisbergi* does not feed exclusively on humans, it is likely that zoonotic transmissions also occur in this CL focus. This significant preference for human hosts (67.57%) further suggested possible transmission of *L*. *tropica* and possibly *L*. *major* in the study area. Rock hyraxes were the second largest bloodmeal sources constituting exclusively 13.51% of sandfly bloodmeals. Presence of rock hyraxes in close proximity to humans and in abundance appears to provide an alternative source of bloodmeal which aids in the amplification of *Leishmania* spp. within the sandflies [57]. Among the 10 sandflies that fed purely on rock hyraxes, three were found to be infected with *L*. *tropica*. Moreover, *L*. *tropica* was identified in one sandfly that had blood from rock hyrax and human. These findings may implicate rock hyraxes as reservoirs of *L*. *tropica*, which would corroborate what others have found for *L*. *tropica* [10,15,58].

Other vertebrate hosts identified in *Ph*. *guggisbergi* bloodmeals included; goats, wild rabbits, wild pigs, rats and mouse. Further investigations are needed to elucidate the potential role of these vertebrates as reservoirs of CL parasites, especially in this complex rural-urban interface where there is unrestricted movement between domestic animals and wildlife. Although the *CYT-B* real-time PCR-HRM successfully identified samples with mixed bloodmeals, it was limited to only those that showed identical peaks to the known controls. In the case where controls are difficult to find, techniques that could allow for the identification of unknown hosts in mixed bloodmeals are recommended to supplement the *CYT-B* real-time PCR-HRM identifications.

Although we identified rock hyrax bloodmeal in one uninfected *Sergentomyia* spp., sandflies of this genus are known to be refractory to *Leishmania* parasites that are pathogenic to humans [59]. However, because they can feed on humans, it is important to control their biting nuisance.

### Conclusion

In this study, we have demonstrated that *Ph*. *guggisbergi* is the most abundant sandfly species distributed across Gilgil sub-county. The high infection rates of *L*. *tropica* in sandflies confirmed this parasite as the predominant *Leishmania* species circulating in the area. Furthermore, high infection rates of *L*. *tropica* in *Ph*. *guggisbergi* that feeds predominantly, but not exclusively, on humans confirmed this species as the main vector of the parasite in Gilgil area. Identification of *L*. *major* infections in *Ph*. *guggisbergi* by real-time PCR suggested this sandfly species as a potential permissive vector of *L*. *major*, a finding that needs to be investigated further. Isolation of live *L*. *tropica* parasites from *Ph*. *saevus sensu lato* indicated this sandfly as a potential vector of *L*. *tropica* which requires further investigations. Sandfly host preference analysis revealed the possibility of zoonotic transmissions of *L*. *tropica* in Gilgil since the main vector does not feed exclusively on humans but also other vertebrates. The potential role of other vertebrate species as reservoirs of *L*. *tropica* and *L*. *major* needs to be explored.

## Supporting information

S1 Fig Sandfly species distribution across the sampling sites in Gilgil. The map was drawn using ArcGIS Online version.
(TIF)

S2 Fig *Leishmania* parasite identification by nested *kDNA*-PCR. M: 100 bp ladder;1 and 2: *Leishmania* spp. isolated from sandflies; 3 and 4: *L*. *major* (Friedlin str.) and *L*. *tropica* positive controls (Lv357); 5: negative control.
(TIF)

S3 Fig Dissociation curves of *L*. *tropica* and *L major* promastigotes mixed in different proportions compared to those of pure *L*. *major* and *L*. *tropica* controls. The proportions were obtained by mixing different volumes of equimolar DNA extracted from *L*. *major* (Friedlin str.) and *L*. *tropica* (Lv357).
(TIF)

S4 Fig *ITS1* gene sequence showing superimposed peaks in the sample exhibiting *L*. *major* and *L*. *tropica* coinfections. Sequencing was done under the forward primer by the Sanger method.
(TIF)
