# Supplementary figures and images for: Association of Phlebotomus guggisbergi with Leishmania major and Leishmania tropica in a complex transmission setting for cutaneous leishmaniasis in Gilgil, Nakuru county, Kenya
Source: PLoS Negl Trop Dis. 2019 Oct 18;13(10):e0007712. doi: 10.1371/journal.pntd.0007712 (PMC6821134; doi:10.1371/journal.pntd.0007712)

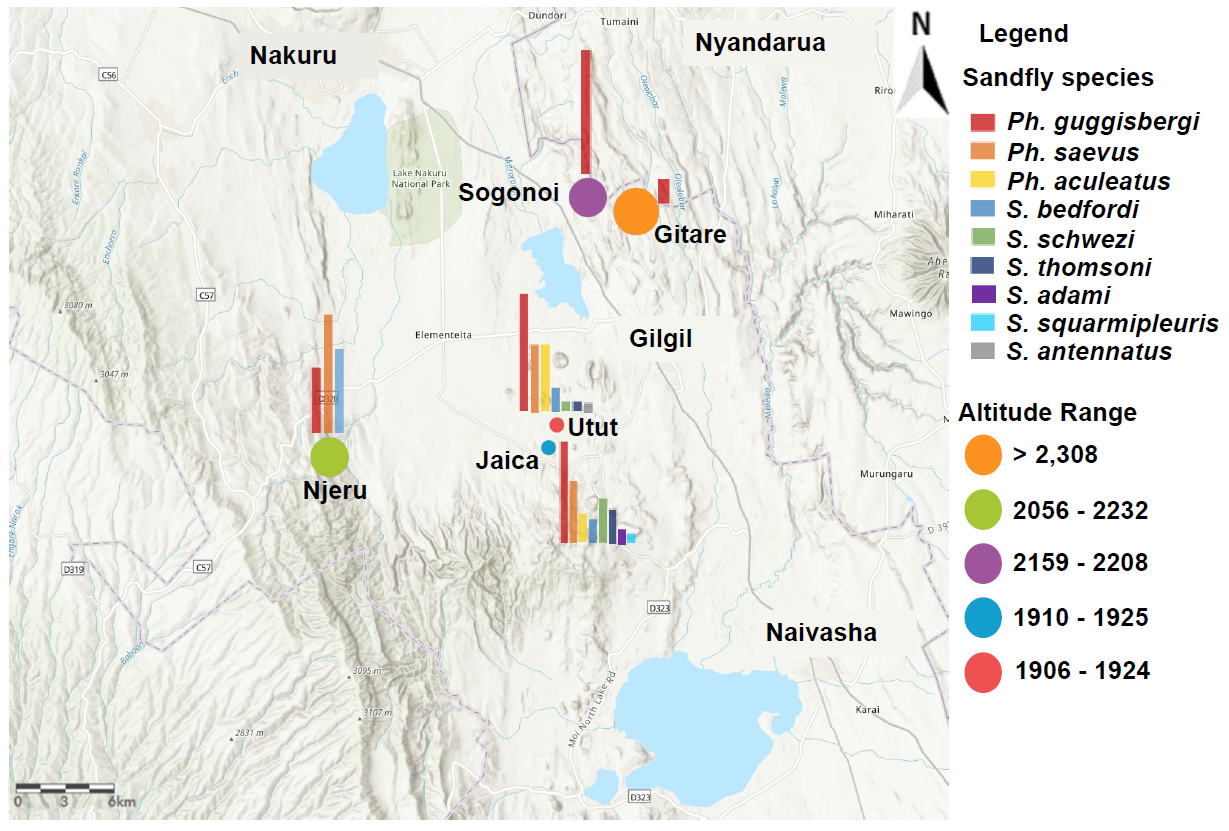

Supplement: S1 Fig — The map was drawn using ArcGIS Online version. (TIF) [file pntd.0007712.s001.tif]

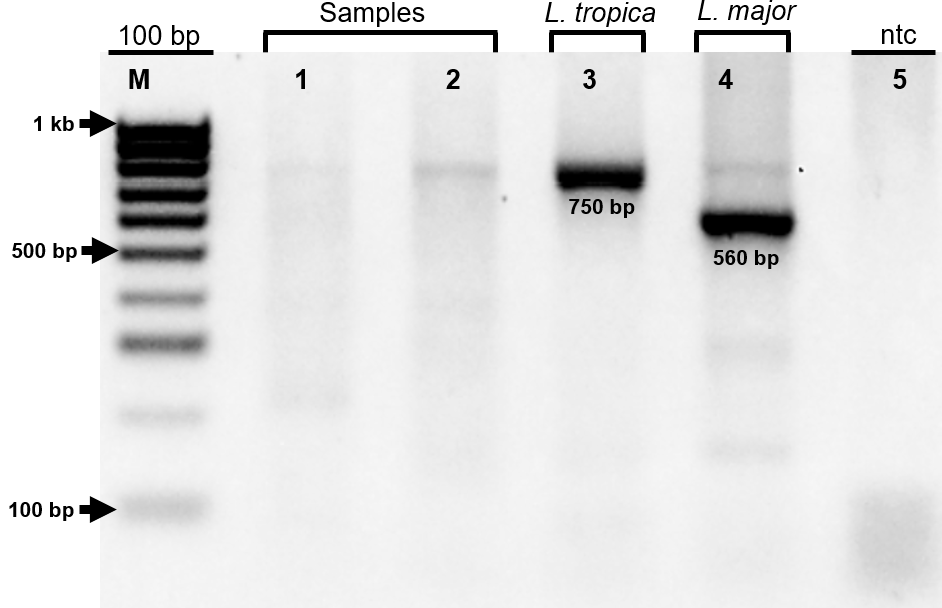

Supplement: S2 Fig — M: 100 bp ladder;1 and 2: Leishmania spp. isolated from sandflies; 3 and 4: L. major (Friedlin str.) and L. tropica positive controls (Lv357); 5: negative control. (TIF) [file pntd.0007712.s002.tif]

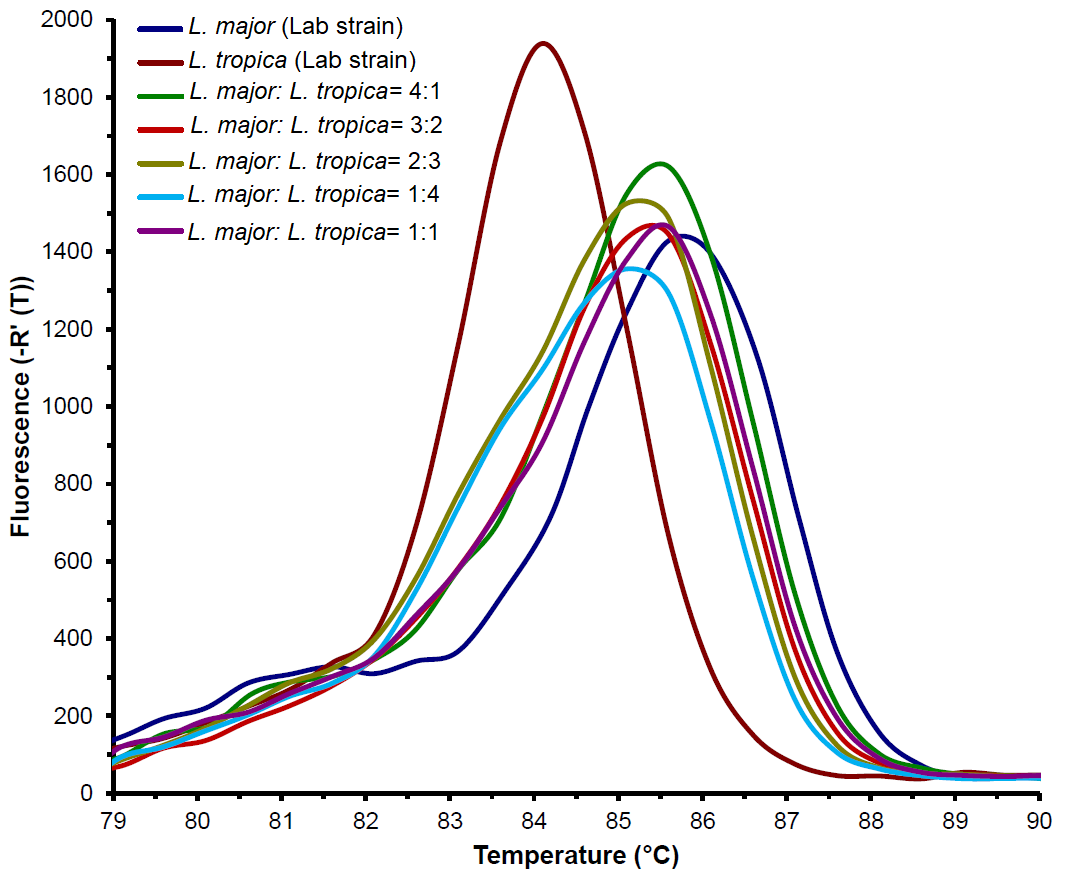

Supplement: S3 Fig — The proportions were obtained by mixing different volumes of equimolar DNA extracted from L. major (Friedlin str.) and L. tropica (Lv357). (TIF) [file pntd.0007712.s003.tif]

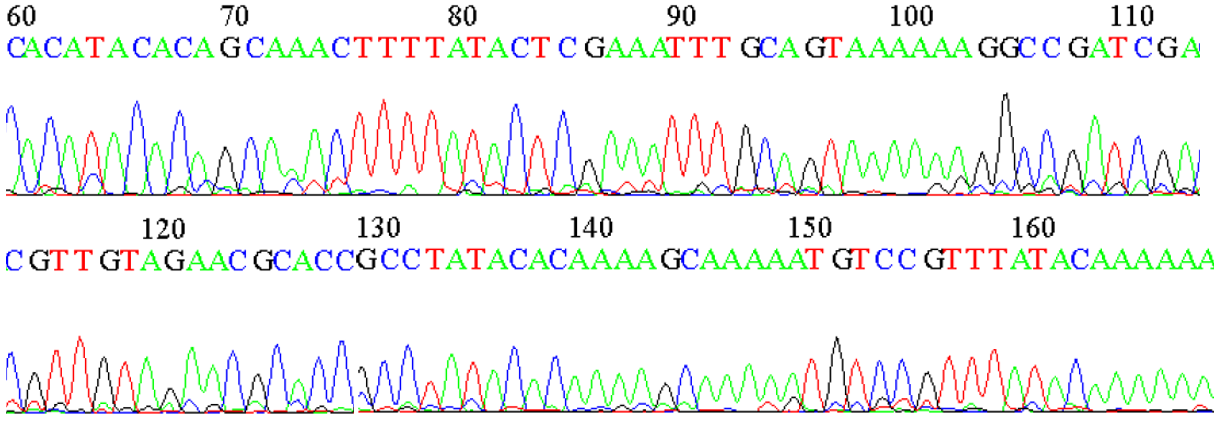

Supplement: S4 Fig — Sequencing was done under the forward primer by the Sanger method. (TIF) [file pntd.0007712.s004.tif]
